# Effects of In Vitro Low Oxygen Tension Preconditioning of Adipose Stromal Cells on Their In Vivo Chondrogenic Potential: Application in Cartilage Tissue Repair

**DOI:** 10.1371/journal.pone.0062368

**Published:** 2013-04-30

**Authors:** Sophie Portron, Christophe Merceron, Olivier Gauthier, Julie Lesoeur, Sophie Sourice, Martial Masson, Borhane Hakim Fellah, Olivier Geffroy, Elodie Lallemand, Pierre Weiss, Jérôme Guicheux, Claire Vinatier

**Affiliations:** 1 INSERM (Institut National de la Santé et de la Recherche Médicale), Unit 791, Center for Osteoarticular and Dental Tissue Engineering, Group STEP “Skeletal Tissue Engineering and Physiopathology”, Nantes, France; 2 University of Nantes, UFR Odontology, Nantes, France; 3 Center for Preclinical Research and Investigation of the ONIRIS Nantes-Atlantic College of Veterinary Medicine, Food Science and Engineering (CRIP), Nantes, France; 4 College of Veterinary Medicine of Nantes (ONIRIS), Department of Equine Surgery, Nantes, France; National University of Ireland, Galway, Ireland

## Abstract

**Purpose:**

Multipotent stromal cell (MSC)-based regenerative strategy has shown promise for the repair of cartilage, an avascular tissue in which cells experience hypoxia. Hypoxia is known to promote the early chondrogenic differentiation of MSC. The aim of our study was therefore to determine whether low oxygen tension could be used to enhance the regenerative potential of MSC for cartilage repair.

**Methods:**

MSC from rabbit or human adipose stromal cells (ASC) were preconditioned *in vitro* in control or chondrogenic (ITS and TGF-β) medium and in 21 or 5% O_2_. Chondrogenic commitment was monitored by measuring *COL2A1* and *ACAN* expression (real-time PCR). Preconditioned rabbit and human ASC were then incorporated into an Si-HPMC hydrogel and injected (i) into rabbit articular cartilage defects for 18 weeks or (ii) subcutaneously into nude mice for five weeks. The newly formed tissue was qualitatively and quantitatively evaluated by cartilage-specific immunohistological staining and scoring. The phenotype of ASC cultured in a monolayer or within Si-HPMC in control or chondrogenic medium and in 21 or 5% O_2_ was finally evaluated using real-time PCR.

**Results/Conclusions:**

5% O_2_ increased the *in vitro* expression of chondrogenic markers in ASC cultured in induction medium. Cells implanted within Si-HPMC hydrogel and preconditioned in chondrogenic medium formed a cartilaginous tissue, regardless of the level of oxygen. In addition, the 3D *in vitro* culture of ASC within Si-HPMC hydrogel was found to reinforce the pro-chondrogenic effects of the induction medium and 5% O_2_. These data together indicate that although 5% O_2_ enhances the *in vitro* chondrogenic differentiation of ASC, it does not enhance their *in vivo* chondrogenesis. These results also highlight the *in vivo* chondrogenic potential of ASC and their potential value in cartilage repair.

## Introduction

Articular cartilage is an avascular and poorly cellularized tissue that has a limited capacity for self-repair after injury. Indeed, only full-thickness defects, which affect both the subchondral bone and cartilage exhibit a repair process that leads to the formation of fibrocartilage. This fibrocartilage does not however display the mechanical properties of native articular cartilage [1] and unfortunately degrades rapidly. This degradation may progress into a premature wear of cartilage and often leads to degenerative joint disease. Different surgical strategies are currently considered such as microfracture [2] or mosaicplasty [3]. For the treatment of cartilage defects, none of these techniques results in a complete regeneration of cartilage tissue [4]. To address this clinical issue, autologous chondrocyte transplantation (ACT) initially developed by Brittberg *et al*. has been introduced into clinical use to treat focal lesions of the knee joint [5], [6], [7]. Given the limitations of autologous chondrocytes (lack of availability and dedifferentiation during amplification), the use of multipotent stromal cells (MSC) for cartilage tissue engineering has recently attracted growing interest [8], [9], [10]. Among the various tissues from which MSC can be isolated, bone marrow has been the most widely used in cartilage repair strategies [11], [12], [13]. However, adherent cells isolated from stromal vascular fraction of adipose tissue also exhibit the major characteristics of stemness (proliferation, long-term self-renewal, and multilineage differentiation) [14] and were named adipose stromal cells (ASC) accordingly [15].

Interestingly, adipose tissue stromal vascular fraction contains 10- to 100-fold more clonogenic cells than bone marrow [16], [17], [18] and is easily accessible through non-invasive liposuction. These practical advantages make ASC an attractive cell population for use in cartilage repair.

Cartilage repair strategies combining MSC and biomaterials have been thoroughly explored recently [10], [19], [20], [21], [22]. In addition to providing a vehicle for the delivery of cells, biomaterials supply a three-dimensional environment suitable for the chondrogenesis of MSC [23], [24].

The use of *in vitro* differentiated MSC for biomaterial-assisted cartilage repair, as opposed to undifferentiated MSC, results in faster and improved tissue repair [25], [26]. However, despite recent progress in understanding MSC biology, the chondrogenic differentiation of MSC remains difficult to control. For this reason, research teams have focused on developing effective culture methods to optimize the chondrogenesis of MSC. While the use of growth factors (such as TGF, BMPs and IGF) for the chondrogenic differentiation of MSC has been widely explored [27], [28], the use of environmental factors, such as oxygen tension, has only recently been contemplated [29].

As mentioned above, cartilage is an avascular tissue in which chondrocytes experience low oxygen tension [30], [31], [32], ranging from 2 to 7% O_2_. Several studies report converging data indicating that low oxygen tension could enhance the chondrogenic differentiation of bone marrow-derived MSC in the presence of induction medium [33], [34]. Of particular interest is Merceron *et al*.'s finding that 5% O_2_ promotes the chondrogenesis of ASC [35]. These data together suggest that low oxygen tension contributes to controlling the chondrogenic commitment and differentiation of various types of progenitor cells including ASC. However, despite a large body of evidence on the *in vitro* prochondrogenic effects of low oxygen tension, it remains unknown whether chondrogenic commitment under low oxygen tension may affect the formation of cartilaginous tissue *in vivo*.

Therefore, the aim of the present study was to determine whether low oxygen tension could be used to enhance the regenerative potential of MSC for cartilage repair. For this purpose, we first assessed the impact of *in vitro* preconditioning with low oxygen tension of ASC on their *in vivo* chondrogenic potential. Next, we investigated two complementary models: (i) the repair of rabbit cartilage defects by the transplantation of autologous ASC in a cellulose-based hydrogel (Si-HPMC hydrogel) and (ii) the formation of cartilaginous tissue by subcutaneous transplantation of human ASC in Si-HPMC hydrogel in nude mice.

## Materials and Methods

### Materials

Hydroxypropyl methylcellulose (HPMC) E4M was purchased from Colorcon-Dow chemical (Bougival, France). Glycidoxypropyltrimethoxysilane (3-GPTMS) was obtained from Acros (Geel, Belgium). Cell culture plastic wares were purchased from Corning BV (Schipol-Rijk, The Netherlands). Hank's Balanced Salt Solution (HBSS), Dulbecco's Modified Eagle's Medium–High Glucose (4.5 g/L) (DMEM), Phosphate-Buffered Saline (PBS), penicillin/streptomycin, trypsin-EDTA (0.05%/0.53 mM), Trizol®, L-glutamine and Superscript® III kit were obtained from Invitrogen (Paisley, UK). 4-(2-hydroxyethyl)-1-piperazineethanesulfonic acid (HEPES), type IA crude collagenase, red blood cell lysis buffer, sodium L-ascorbate, Insulin Transferrin Sodium Selenite (ITS) media supplement, dexamethasone, alcian blue, hyaluronidase and type II collagenase (290 units/mg) were purchased from Sigma-Aldrich (St. Louis, MO). TGF-β1 was obtained from PeproTech Inc. (London, UK). NucleoSpin® RNA II was obtained from Macherey-Nagel (Hoerdt, France). Brilliant® SYBR® Green Master Mix was obtained from Stratagene (La Jolla, CA). The PCR primers were synthesized by MWG Biotech (Ebersberg, Germany). Fetal calf serum (FCS) was purchased from Dominique Dutscher (Brumath, France). Technovit 9100 New® was obtained from Heraeus Kulzer (Wehrheim/Ts, Germany). The mouse monoclonal antibody directed against human and rabbit type II collagen was purchased from MP Biomedicals (Solon, OH). The biotinylated goat anti-mouse IgG antibody, the Universal Dako LSAB® (labelled streptavidin biotin reagents) and peroxidase kit were purchased from Dako (Trappes, France). All other chemicals were obtained from standard laboratory suppliers and were of the highest grade of purity available.

### Preparation of Si-HPMC hydrogel

As previously described, Si-HPMC (silanized hydroxypropyl methylcellulose) was synthesized by grafting 14.24% 3-GPTMS onto E4M1 in heterogeneous medium [36]. Si-HPMC powder (3% w/v) was solubilized in 0.2 M NaOH under constant stirring for 48 h. The solution was then sterilized by steam (at 121°C for 20 min). Finally, to allow the formation of a reticulated hydrogel, the solution was mixed with 0.5 volume of 0.26 M HEPES buffer. The final product was a viscous liquid at pH 7.4, which allowed cell incorporation. The cell/Si-HPMC hydrogel mixture was then reticulated for approximately 30 min, as previously described [36].

### Rabbit and mice surgery

Rabbit and mouse handling, as well as surgical procedures, were conducted according to European Community guidelines for the care, accommodation and use of laboratory animals (DE 86/609/CEE; modified DE 2003/65/CE). Experiments were performed according to good laboratory practices at the Center for Preclinical Research and Investigation of the ONIRIS Nantes-Atlantic College of Veterinary Medicine, Food Science and Engineering. All rabbit experimental studies were performed on adult female New Zealand White rabbits weighing 3 to 3.5 kg (Charles River, L′Arbresle, France). All mouse experimental studies were performed on 1-month-old female Swiss nude mice (Charles River, L′Arbresle, France). General anesthesia of rabbits was induced by intramuscular injection of ketamine (0.5 mL/kg, Imalgene 1000®, Merial SAS, France) and xylazine (0.3 mL/kg, Rompun®, Bayer, France) cocktail.

Intravenous injections were carried out to extend intramuscular administration until effects at one tenth of the initial intramuscular dosage and repeated on demand, once or twice during the whole surgical period.

Pre-operative analgesia was provided through subcutaneous injection of morphine chlorhydrate (2 mg/kg). Immediate post-operative analgesia was provided through subcutaneous injection of meloxicam (0.1 mg/kg, Metacam®, Boehringer Ingelheim, France), and prolonged for 5 days orally. Rabbits were euthanized by intra-cardiac injection of 5 mL of pentobarbital (Dolethal®, Vetoquinol S.A., France) after inducing general anesthesia as described above.

Mice were pre-medicated with morphine chlorhydrate (2 mg/kg) diluted into sterile saline solution and injected subcutaneously. General anesthesia was obtained in an induction chamber with isoflurane (2%) delivered in O_2_ and prolonged through an individual mask. Mice were euthanized by an overdose of isoflurane within an induction chamber.

### Isolation, expansion and chondrogenic differentiation of rabbit and human adipose stromal cells

ASC were obtained from human patients (hASC) undergoing liposuction and who had given written consent (ethics committees: Agence de BioMedecine, n°PFS08-018, the legislation code L.1211-3 toL.1211-9: residues obtained during a surgical procedure, performed in the interest of the person operated, can be used for scientific research), or from autologous rabbit adipose tissue (rASC) harvested from the inguinal region. Briefly, and as previously described [35], human lipoaspirate and rabbit adipose tissue were shredded into small pieces and washed extensively with HBSS. The washed adipose tissue was treated with collagenase (0.025%) in HBSS for 1 h at 37°C under gentle agitation. The collagenase was inactivated by adding an equal volume of DMEM containing 1% penicillin/streptomycin, 1% L-glutamine and 10% FCS (control medium). The digested product was then centrifuged at 250 g for 5 min to separate adipose fraction from stromal fraction. The supernatant was removed and the stromal cells were re-suspended in the control medium and filtered through a 70 µm nylon mesh filter. The filtrate was centrifuged and the cells were re-suspended in red blood cell lysis buffer. The lysis reaction was stopped by adding control medium. The suspension was centrifuged and the cells were finally re-suspended in control medium and plated at a density of 5×10^4^ cells/cm^2^.

hASC isolated using the protocol described above have been extensively characterized in our laboratory (for details see [35], [37]).

The medium was replaced 24 h after seeding to remove non-adherent cells. To prevent spontaneous differentiation, primary cultures (P0) of ASC were grown to approximately 80% of confluence and then detached from the cell culture flask using trypsin-EDTA. For all subsequent experiments, ASC at passage 2 were used.

All culture incubations were performed at 37°C in a humidified atmosphere containing 5% CO_2_ and the medium was changed every 2 to 3 days.

For *in vitro* chondrogenic differentiation, ASC were divided into three experimental groups. The cells were cultured for 21 days in monolayers (1×10^4^ cells/cm^2^) under normoxic conditions (21% O_2_) in control medium (NCT) or in chondrogenic medium (NCH); otherwise, they were cultured under hypoxic conditions (5% O_2_) in chondrogenic medium (HCH). The chondrogenic medium was composed of serum-free DMEM supplemented with 1% penicillin/streptomycin, 6.25 µg/mL insulin, 6.25 µg/mL transferrin, 6.25 ng/mL sodium selenite (ITS), 50 nM sodium L-ascorbate, 1×10^−8^ M dexamethasone and 10 ng/mL TGF-β1 as described previously [35], [38]. For *in vitro* culture under hypoxic conditions, ASC were incubated at 37°C, in a tri-gas incubator (Binder, Tuttlingen, Germany) delivering 5% CO_2_, 5% O_2_ and 90% N_2_ in a humidified atmosphere.

### 3D culture of human adipose stromal cells in Si-HPMC hydrogel

As described previously, hASC were collected and gently mixed with Si-HPMC hydrogel at a density of 2×10^6^ cells/mL of hydrogel [39]. The hASC/Si-HPMC mixture was distributed in 12-well plates (1 mL/well) and incubated at 37°C and 5% CO_2_. After a 2 h-incubation, control medium was added. After 24 h, hASC/Si-HPMC hydrogel constructs were separated into three experimental groups and cultured in NCT, NCH and HCH conditions for 21 days. The media were changed every 2 to 3 days.

### Real-time PCR analysis of the chondrogenic differentiation of rabbit and human adipose stromal cells

Total RNA was extracted from monolayer cultures using a Nucleospin® ARN II kit in accordance with the manufacturer's instructions. For hASC cultured in the 3D Si-HPMC hydrogel, total RNA was extracted with Trizol®. One microgram of total RNA was reverse-transcribed using the Superscript® III kit in a total volume of 20 µL. Complementary DNA (cDNA) was amplified in a total volume of 25 µL of PCR reaction mix containing 12.5 µL of Brilliant® SYBR® Green Master Mix (1X), 30 nM SYBR green reference dye and each primer at a concentration of 10 µM. The sequences of the rabbit and human primers are provided in Tables 1 and 2, respectively. The COL2A1 gene encodes the alpha-1 chain of type II collagen, a fibrillar collagen found specifically in cartilage. The ACAN gene encodes aggrecan core protein. Aggrecan is the major member of the proteoglycan family found in the extracellular matrix of cartilaginous tissue. Real-time PCR was performed in a MX3000P® real-time PCR system (Stratagene) under the following conditions: 10 min at 95°C followed by 40 cycles of 30 s at 95°C, 1 min at 60°C and 30 s at 72°C. The efficiency and specificity of each primer set was confirmed using standard curves of cycle threshold values vs. serial dilutions of total RNA and by evaluating the melting profile. Cycle thresholds were normalized to those of β-actin, used as the reference gene, to control for differences in cDNA quantification. The results were reported as relative expression levels.

**Table 1:** Sequences of rabbit primer pairs, gene bank accession numbers used for real-time PCR analysis and size of the PCR products.

Gene	Gene Bank Accession Number	Sequence	Base Pairs (bp)
*Atcb* (b-actin)	NM_001101683	Fwd 5′-CCCATCTACGAGGGCTACGC-3′	152
		Rev 5′- TCCTTGATGTCCCGCACGATC-3′	
*Col2a1* (type II collagen)	NM_001195671	Fwd 5′-ACAGCAGGTTCACCTATACCG-3′	60
		Rev 5′-CCCACTTACCGGTGTGTTTC-3′	
*Acan* (aggrecan)	XM_002723376	Fwd 5′-GAGGATGGCTTCCACCAGT-3′	61
		Rev 5′-TGGGGTACCTGACAGTCTGA-3′	

**Table 2:** Sequences of human primer pairs, gene bank accession numbers used for real-time PCR analysis and size of the PCR products.

Gene	Gene Bank Accession Number	Sequence	Base Pairs (bp)
*ACTB* (β-actin)	NM_001101	Fwd 5′- CCAACCGCGAGAAGATGA -3′	97
		Rev 5′- CCAGAGGCGTACAGGGATAG -3′	
*COL2A1* (type II collagen)	NM_001844	Fwd 5′- TGTCAGGGCCAGGATGTC -3′	63
		Rev 5′- ATCATTATACCTCTGCCCATCC -3′	
*ACAN* (aggrecan)	NM_001135	Fwd 5′- CCTCCCCTTCACGTGTAAAA -3′	64
		Rev 5′- GCTCCGCTTCTGTAGTCTGC -3′	

### Implantation of rabbit adipose stromal cells within Si-HPMC hydrogel in rabbit articular defects

After a medial parapatellar incision, the patella was luxated laterally. Two osteochondral defects with a diameter of 3 mm and a depth of 4 mm were created in the patellar groove of the femur, using a surgical round bur on a slow-speed rotary dental handpiece, as described previously [10]. The surgical procedure was performed on both sides. For the implantation of autologous rASC/Si-HPMC hydrogel into articular cartilage defects, cells were individualized by tryspin/EDTA treatment, centrifuged at 250 g for 5 min. Two million individualized rASC were gently mixed with 200 µL of the Si-HPMC hydrogel before its reticulation. Defect sites were filled with the Si-HPMC hydrogel containing autologous rASC preconditioned in NCT, NCH, or HCH conditions. As reported previously [10], autologous rabbit nasal chondrocytes (RNCs), used as the positive control, were implanted at a density of 0.5×10^6^ RNC/200 µL of Si-HPMC hydrogel. Autologous RNCs were isolated from the nasal septum, harvested and cultured, as described previously [10]. The four different conditions (rASC cultured in NCT, NCH, HCH and RNC) were tested in triplicates and three animals received implants (four implants per rabbit; two per patellar groove). After surgery, the animals were allowed to move freely in their cages. After eighteen weeks, rabbits were sacrificed and the samples were histologically processed as described below.

### Implantation of human adipose stromal cells within Si-HPMC hydrogel in nude mice subcutis

hASC were cultured in NCT, NCH and HCH for three weeks and 0.5×10^6^cells were individualized and gently mixed with 250 µL of Si-HPMC hydrogel prior to subcutaneous implantation into nude mice, as described previously [37]. As a control, primary horse nasal chondrocytes (HoNCs) incorporated into Si-HPMC hydrogel (0.5×10^6^ HoNC/250 µL) were injected subcutaneously.

The four different conditions (ASC cultured in NCT, NCH, HCH and HoNCs) were tested in triplicates and six animals received implants (two implants per animal). The animals were sacrificed five weeks after implantation and the samples were processed histologically as described below.

### Histological analysis of explants

A group of rabbit explants was embedded in resin Technovit 9100 New® as described by Yang *et al*. [40] and stained using Movat's pentachrome [41].

The second group of rabbit explants, mice explants and hASC cultured in the Si-HPMC hydrogel were embedded in paraffin and stained or immunostained, as described previously [37], [42]. Briefly, the explants embedded in resin and paraffin were cut in 5 µm-thick sections passing through the middle of the defects in the coronal plane. The production of a cartilaginous matrix containing sulfated glycosaminoglycans (GAG) and type II collagen was evaluated using alcian blue staining and type II collagen immunostaining, respectively. For type II collagen immunostaining, human nasal cartilage sections were used as a positive control. As a negative control, the sections were processed using an identical protocol, but omitting the primary antibody. The sections were then visualized using a light microscope (Zeiss Axioplan2, Göttingen, Germany), with immuno-positive areas exhibiting brown staining.

The histological sections were evaluated by double-blind, randomized scoring performed by five trained, independent examiners for each section (n = 3 per replicate). To evaluate the quality of the repaired tissue in rabbits after surgery, the sections were scored according to O′Driscoll's method [43]. O′Driscoll scoring assesses the nature of the predominant tissue (cellular morphology 0–4; matrix staining 0–3), the structural characteristics (surface regularity 0–3; surface integrity 0–2; thickness 0–2; bonding to the adjacent cartilage 0–2), cellular degenerative changes (hypocellularity 0–3; chondrocyte clustering 0–2) and changes in adjacent cartilage (0–3). The score for a normal cartilage is 24.

### Statistical analysis

Each *in vitro* experiment was repeated at least three times with similar results. Results are expressed as mean ± SEM of triplicate determinations. Means were compared using a one-way ANOVA followed by a post-hoc test (Tukey's honestly significant difference). Histological grading scores were analyzed using the Wilcoxon Mann-Whitney test. A *p*-value <0.05 was considered statistically significant.

## Results

### Chondrogenic potential of differentially preconditioned rabbit adipose stromal cells

**Figure 1:**
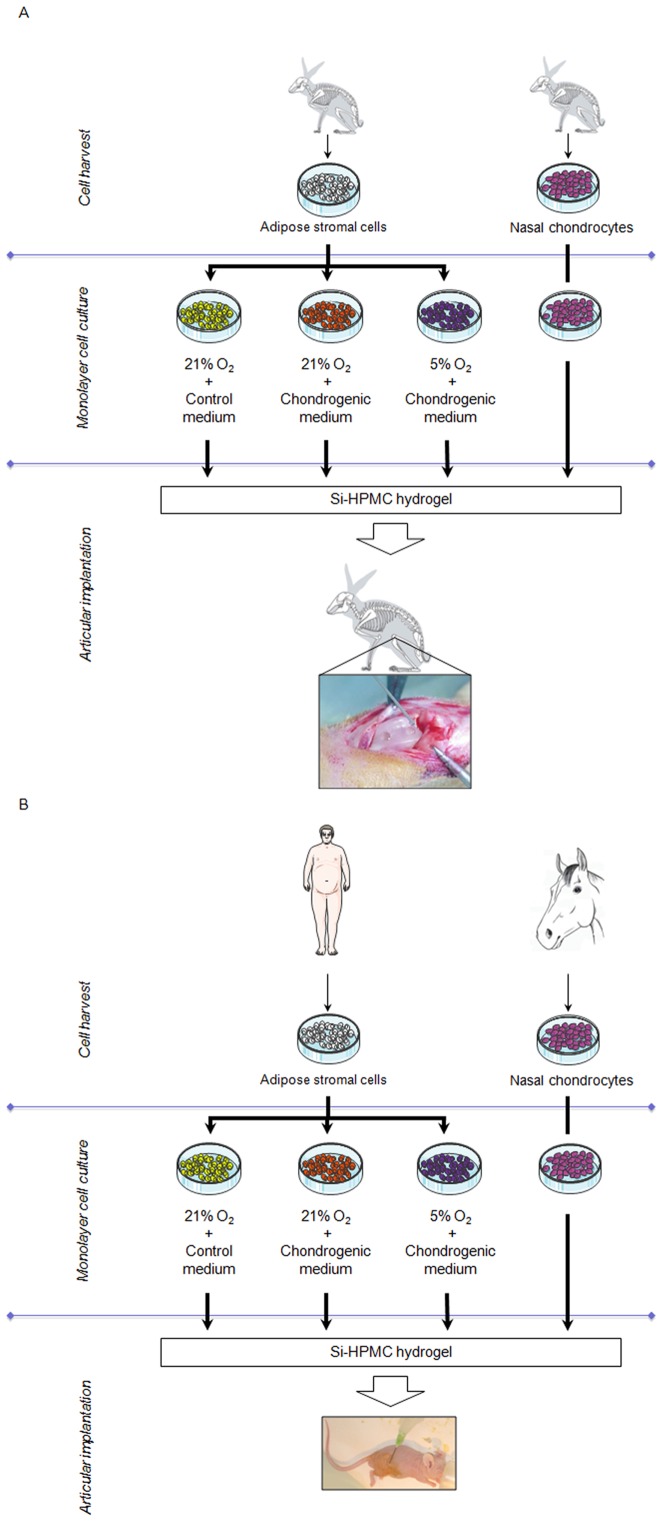
Schematic overview of *in vivo* experimental design. A) Schematic overview of the chondrogenic potential of differentially preconditioned rabbit adipose stromal cells (rASC). rASC were isolated and cultured under normoxic conditions (21% O_2_) in control medium or chondrogenic medium or under hypoxic conditions (5% O_2_) in chondrogenic medium. As a positive control, rabbit nasal chondrocytes (RNC) were used. Preconditioned rASC and RNC were finally associated with Si-HPMC hydrogel and implanted in rabbit articular cartilage defects for 18 weeks. B) Schematic overview of the chondrogenic potential of differentially preconditioned human adipose stromal cells (hASC). hASC were isolated and cultured under normoxic conditions (21% O_2_) in control medium or chondrogenic medium or under hypoxic conditions (5% O_2_) in chondrogenic medium. As a positive control, horse nasal chondrocytes (HoNC) were used. Preconditioned hASC and HoNC were finally associated with Si-HPMC hydrogel and implanted in nude mice subcutis for 5 weeks.

**Figure 2:**
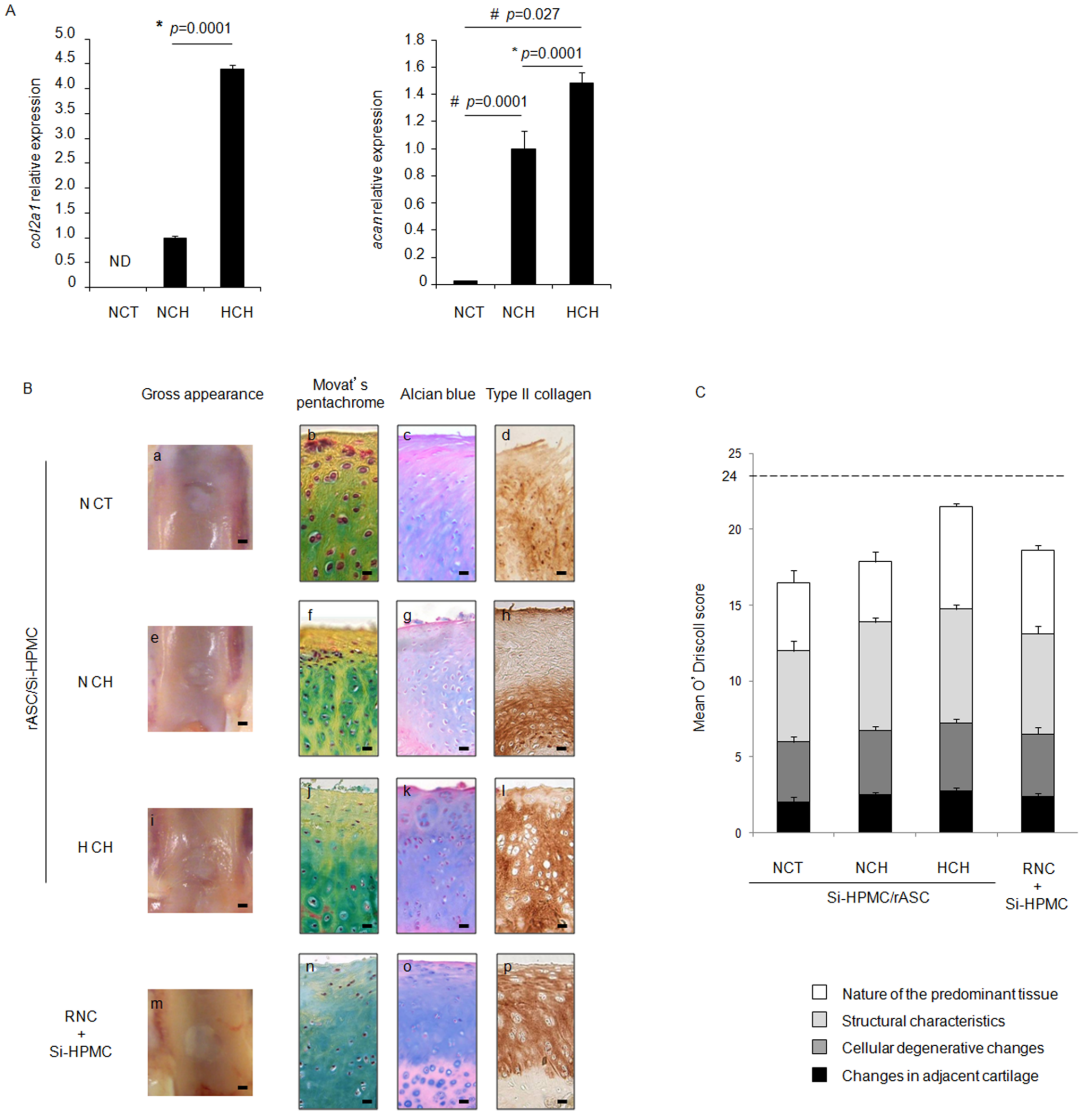
Chondrogenic potential of differentially preconditioned rabbit ASC (rASC). **A)** rASC were cultured under normoxic conditions (21% O_2_) in control medium (NCT) and chondrogenic medium (NCH) or under hypoxic conditions (5% O_2_) in chondrogenic medium (HCH). The expression of transcripts encoding type II collagen (*col2a1*) and aggrecan (*acan*) was measured by real-time PCR. The results are expressed as relative expression levels. ND: not detected. # *p*<0.05 compared with NCT; * *p*<0.05 compared with NCH. **B)** rASC were cultured in NCT (a, b, c, d), NCH (e, f, g, h), or HCH (i, j, k, l) and implanted with the Si-HPMC hydrogel in rabbit osteochondral defects. Rabbit nasal chondrocytes (RNCs) incorporated into the Si-HPMC hydrogel were used as a control (m, n, o, p). After 18 weeks of implantation, the defects were macroscopically observed [gross appearance (a, e, i, m)], histologically stained using Movat's pentachrome (b, f, j, n) and alcian blue (c, g, k, o) and immunostained for type II collagen (d, h, l, p). a, e, i, m: bar indicates 1 mm. b–d; f–h, j–l, n–p: bar indicates 100 µm. **C)** A semi-quantitative analysis of the regenerated tissue was performed using O′Driscoll's repair score as described in the “Materials and Methods” section. The results are expressed as a mean O′Driscoll score.

Prior to investigating the *in vivo* effects of hypoxic preconditioning of autologous rASC, we characterized the *in vitro* phenotype of differentially preconditioned rASC. The rASC were cultured in a monolayer under NCT, NCH, or HCH conditions (Fig. 1A). Our real-time PCR data indicate that, in the NCT condition, the expression of type II collagen (*col2a1*) mRNA could not be detected (ND) and aggrecan (*acan*) mRNA was barely detectable. The expression of *col2a1* and *acan* became detectable in the NCH condition and substantially increased in rASC cultured in HCH conditions with significant 4.5- and 1.6-fold increases, respectively, compared with the NCH condition (Fig. 2A).

We next aimed to determine the effects of *in vitro* hypoxic preconditioning of rASC on their *in vivo* chondrogenic potential. rASC were cultured in the three conditions mentioned above and implanted within a Si-HPMC hydrogel in rabbit articular cartilage defects.

The newly formed tissue after implantation of the differentially preconditioned rASC/Si-HPMC hydrogel was first histologically characterized. Movat's pentachrome staining (Fig. 2B b, f, j) revealed yellow collagen fibers, especially in the superficial zone, in the NCT and NCH conditions. Round/oval cells and green/blue stained GAG seemed to be more predominant in the middle and deep zones. Alcian blue staining (Fig. 2B c, g, k) and immunostaining for type II collagen (Fig. 2B d, h, l) revealed the presence of sulfated GAG and type II collagen in the three conditions. In the NCT condition, GAG was weakly stained and immunostaining for type II collagen remained slight. When rASC were preconditioned in the NCH condition, alcian blue staining remained weak and type II collagen was mainly noted in the deep zone of the newly formed tissue. For rASC preconditioned in the HCH condition, GAG and type II collagen were homogenously detected.

As expected, the implantation of the autologous RNC/Si-HPMC hydrogel induced the formation of a well-organized tissue (Fig. 2B n) rich in GAG (Fig. 2B o) and type II collagen (Fig. 2B p).

To further analyze the newly formed tissue, a semi-quantitative assessment was performed (Fig. 2C) using O′Driscoll's score. No difference between NCT- and NCH-preconditioned rASC and RNC was noted (16.5±0.1; 17.9±0.1; and 18.6±0.065, respectively). The score for HCH-preconditioned rASC/Si-HPMC (21.5±0.01) was slightly, but not significantly, higher when compared with the other conditions.

The histological analyses revealed that the implantation of differentially preconditioned rASC/Si-HPMC hydrogel constructs led to the formation of a repair tissue containing GAG and type II collagen to a similar extent, regardless of the type of preconditioning used. Thus, although low oxygen tension exerts an *in vitro* pro-chondrogenic effect, the *in vivo* articular environment could overcome this effect.

### Chondrogenic potential of differentially preconditioned human adipose stromal cells

To counteract this potential effect of the articular environment and with the long-term goal of developing a human therapy, we next tested the subcutaneous implantation of human ASC in nude mice (Fig. 1B).

**Figure 3:**
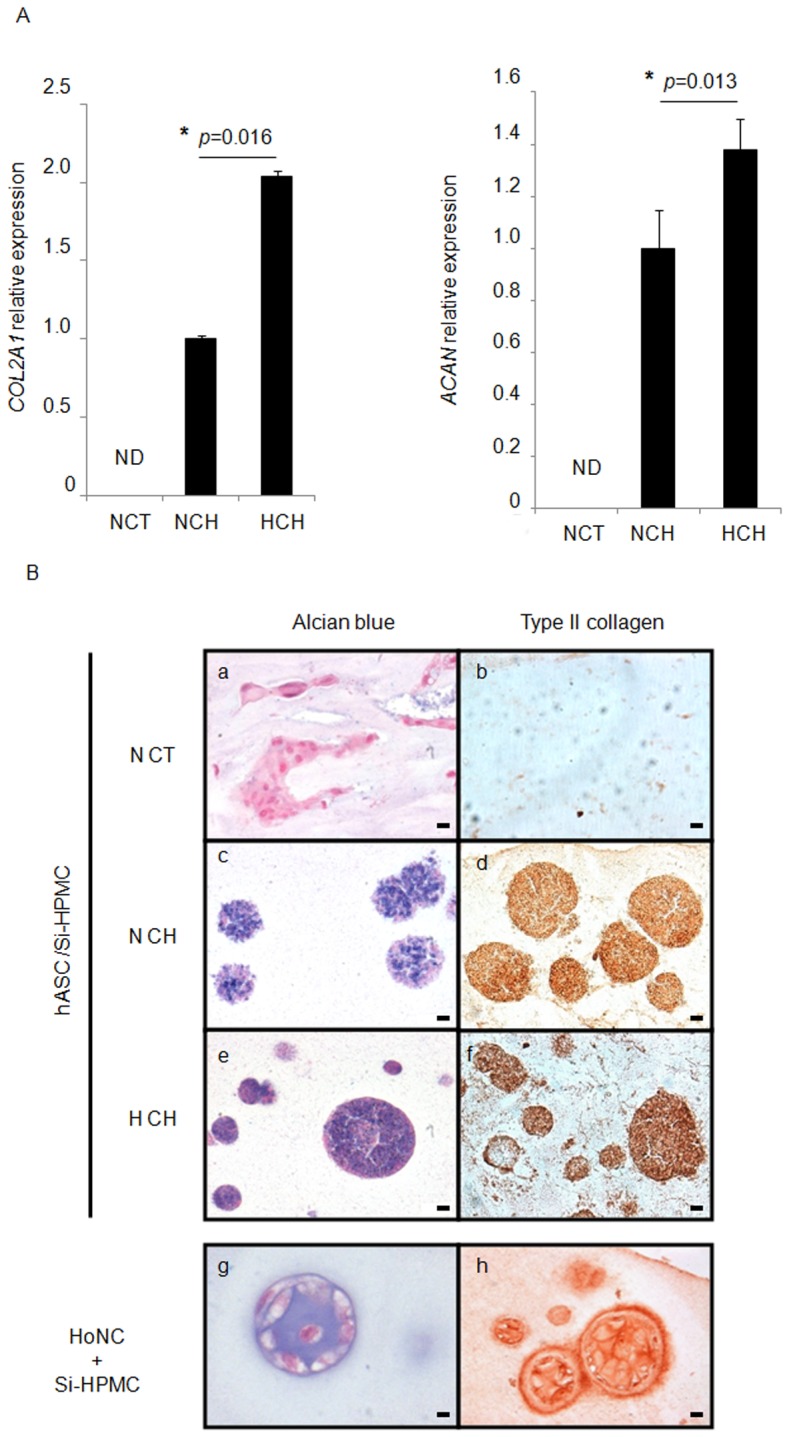
Chondrogenic potential of differentially preconditioned human ASC (hASC). **A)** hASC were cultured under normoxic conditions (21% O_2_) in control medium (NCT) and chondrogenic medium (NCH) or under hypoxic conditions (5% O_2_) in chondrogenic medium (HCH). The expression of transcripts encoding type II collagen (*COL2A1*) and aggrecan (*ACAN*) was measured using real-time PCR. The results are expressed as relative expression levels. ND: not detected * *p*<0.05 compared with NCH. **B)** hASC were cultured in NCT (a, b), NCH (c, d) or HCH (e, f) and implanted with the Si-HPMC hydrogel into subcutaneous pockets of nude mice. Horse nasal chondrocytes (HoNCs) incorporated into the Si-HPMC hydrogel were used as a control (g, h). After five weeks, the samples were harvested, histologically stained using alcian blue (a, c, e, g) and immunostained for type II collagen (b, d, f, h). Bar indicates 20 µm.

Before investigating the impact of hypoxic preconditioning of hASC on their *in vivo* chondrogenic potential, the phenotypes of differentially preconditioned hASC were first characterized. Our real-time PCR data revealed that the expression levels of *COL2A1* and *ACAN* mRNA could be detected only for cells cultured in NCH and HCH (Fig. 3A). The mRNA for these genes was significantly upregulated 2- and 1.3-fold in chondrogenic medium under hypoxic conditions compared with normoxic conditions, respectively. Similar to the rASC findings, these results confirm that an induction medium is required for the induction of type II collagen and aggrecan expression and that 5% O_2_ increases their expression.

To address the effects of hypoxic preconditioning on the chondrogenic potential of hASC *in vivo*, differentially preconditioned hASC were incorporated into Si-HPMC hydrogel and injected into subcutaneous pockets of nude mice. The histological examination of the newly formed tissue using NCT-preconditioned hASC revealed the absence of cell aggregate formation (Fig. 3B a, b). In contrast, hASC implants that had been preconditioned in NCH or HCH revealed the formation of cell aggregates (Fig. 3B c, d, e, f) that were positively stained by alcian blue and immunoreactive for type II collagen, thus suggesting the production of a cartilaginous matrix. As expected, primary HoNCs used as the positive control revealed the formation of cartilage-like aggregates containing GAG and type II collagen (Fig. 3B g, h).

Although low oxygen tension exerts an *in vitro* prochondrogenic effect, our data reveal that hASC cultured in chondrogenic medium, regardless of oxygen tension, are able to form cartilaginous cell aggregates to a similar extent.

These findings suggest that Si-HPMC may be a suitable scaffolding hydrogel that allows cells to adequately sense their environment.

### *In vitro* chondrogenic differentiation of 3D-cultured human adipose stromal cells

**Figure 4:**
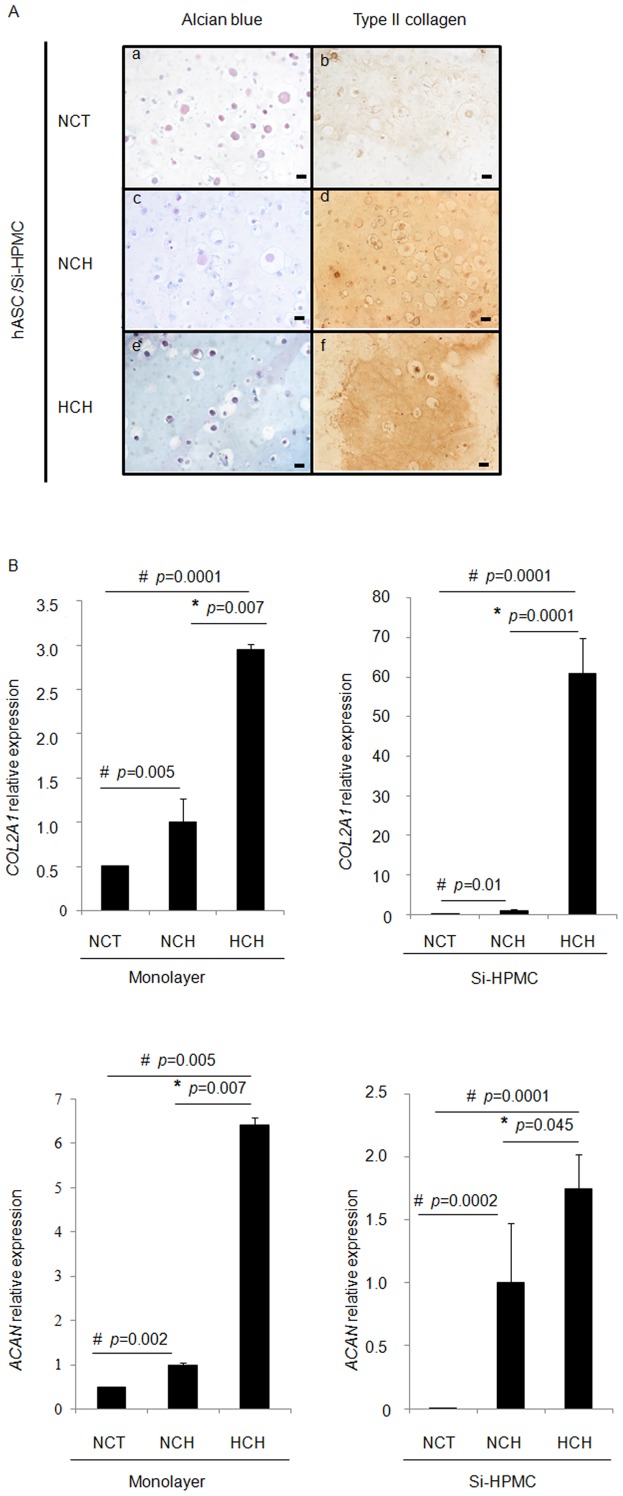
Chondrogenic differentiation of 3D cultured human ASC (hASC). **A)** hASC were 3D cultured within the Si-HPMC hydrogel under normoxic conditions (21% O_2_) in control medium (NCT) (a, b) and chondrogenic medium (NCH) (c, d) or under hypoxic conditions (5% O_2_) in chondrogenic medium (HCH) (e, f). The presence of sulfated glycosaminoglycans and type II collagen was investigated using alcian blue staining (a, c and e) and type II collagen immunostaining (b, d and f), respectively. Bar indicates 20 µm. **B)** hASC were cultured in a monolayer or within the Si-HPMC hydrogel under normoxic conditions (21% O_2_) in control medium (NCT) and chondrogenic medium (NCH) or under hypoxic conditions (5% O_2_) in chondrogenic medium (HCH). The expression of transcripts encoding type II collagen (*COL2A1*) and aggrecan (*ACAN*) was measured by real-time PCR. The results are expressed as relative expression levels. ^#^ *p*<0.05 compared with NCT. * *p*<0.05 compared with NCH.

To address whether ASC cultured within the Si-HPMC hydrogel respond to a prochondrogenic environment (i.e., 3D culture, chondrogenic medium and low oxygen tension), hASC were cultured within Si-HPMC hydrogel in NCT, NCH and HCH conditions. The *in vitro* production of a cartilaginous matrix was evaluated by alcian blue staining and type II collagen immunostaining. hASC cultured in NCT/Si-HPMC hydrogel exhibited weak alcian blue staining and type II collagen immunostaining (Fig. 4A a, b). In contrast, hASC cultured in chondrogenic medium within the Si-HPMC hydrogel were positive for GAG and type II collagen, especially when cultured under 5% O_2_ (Fig. 4A c–f).

To further evaluate the scaffolding properties of the Si-HPMC hydrogel, we compared the expression of *COL2A1* and *ACAN* mRNA in hASC cultured in monolayer or within the Si-HPMC hydrogel under the NCT, NCH and HCH conditions. According to the results obtained by real-time PCR, hASC cultured in a monolayer in NCT or in the NCT/Si-HPMC hydrogel barely expressed the two transcripts. In the monolayer condition, the chondrogenic medium induced a 2-fold increase in the expression of these transcripts, when compared with the NCT condition. In the Si-HPMC hydrogel condition, the chondrogenic medium induced 8- and 125-fold increases in the expression of *COL2A1* and *ACAN* mRNA, respectively, when compared with the NCT condition (Fig. 4B).

In addition, a 3- and 6-fold increase in *COL2A1* and *ACAN* transcripts, respectively, was observed in hASC cultured in the HCH monolayer, when compared with the NCH/monolayer. In Si-HPMC hydrogel, the expression of *COL2A1* and *ACAN* was increased by 60- and 1.5-fold, respectively, for hASC cultured in HCH compared to those cultured in NCH.

These results suggest that hASC cultured within Si-HPMC hydrogel are responsive to a prochondrogenic medium and a 5% O_2_ tension. In addition, our data strongly suggest that a 3D culture within Si-HPMC hydrogel may support the capacity of the prochondrogenic condition to enhance the chondrogenic differentiation of hASC.

## Discussion

In the last decade, MSC-based regenerative strategies have been thoroughly investigated for the formation of long-term functional tissue in cartilage repair. However, controlling the chondrogenic commitment and differentiation of MSC remains challenging [44]. Among the various chondrogenic factors that could be used to exploit the potential of MSC for cartilage regeneration, hypoxia is probably among the most tunable, safe and easy-to-use. In this context, we evaluated whether *in vitro* low oxygen tension could impact the cartilage regenerative potential of ASC after *in vivo* implantation.

Consistent with our previously published data [35], our *in vitro* results confirmed that low oxygen tension increased the expression of the two major chondrogenic markers in monolayer-cultured ASC of rabbit and human origin. This first set of experiments also allowed us to determine whether ASC exhibited different levels of chondrogenic commitment after *in vitro* preconditioning under various conditions (NCT, NCH and HCH), especially at the mRNA level.

Next, we evaluated the *in vivo* chondrogenic potential of these differentially preconditioned ASC. To enable the *in vivo* implantation of ASC, we used an injectable and self-setting cellulose-based hydrogel (Si-HPMC) that was developed for skeletal tissue engineering [42]. We then performed *in vivo* experiments in two complementary animal models that are widely used in cartilage tissue engineering: the repair of osteochondral defects in the rabbit knee joint [41] and the formation of subcutaneous cartilaginous cell aggregates in nude mice [37].

Based on our histological data and regardless of the preconditioning conditions, rabbit ASC were found to generate repair tissue in cartilage defects. It is well known, however, that the functional load-bearing capacity of cartilaginous repair tissue is dependent on the ultrastructural components and the organization of the newly formed tissue [45], [46]. On the one hand, vertical collagen fibers in the deep zone of the cartilage counteract swelling and protect the extracellular matrix from strain at the subchondral junction. On the other hand, horizontal fibers in the superficial zone play a critical role in tangential resistance to shear stress at the articular surface. Given the biomechanical relevance of this specific histological organization of hyaline cartilage, it was particularly notable in the present study that preconditioned ASC, especially in chondrogenic medium and hypoxic conditions, induced the formation of repair tissue that exhibited a hyaline-like organization. These data confirm the potential of ASC for cartilage engineering.

Surprisingly, although 5% oxygen tension dramatically stimulated the *in vitro* chondrogenic differentiation of rASC, it failed to significantly enhance the *in vivo* formation of cartilage-like tissue in the rabbit articular site.

However, a crucial point when interpreting the results from the *in vivo* cartilage repair experiments is determining how much the cells actually influenced the outcome, as spontaneous repair is known to occur in osteochondral defects [47].

Therefore, the repair of an osteochondral defect in rabbits would probably not constitute the most relevant model to accurately evaluate the regenerative potential of cells. Thus, to counteract the endogenous regenerative effects of the articular environment, we implanted human ASC in nude mice subcutis in one of the most widely established models used to decipher the regenerative potential of cell biomaterial constructs.

In this model, and in contrast to the effect observed for NCT-preconditioned cells, chondrogenically induced human ASC incorporated into Si-HPMC hydrogel formed cartilage-like cell aggregates enriched in type II collagen and GAG. However, as previously reported for rabbit knee joints, 5% low oxygen tension did not stimulate the formation of cartilage-like aggregates. In contrast to the data obtained using rabbit ASC in the cartilage defect model, the findings in the subcutaneous nude mouse model highlight the beneficial effect of the induction medium on the *in vivo* chondrogenesis of hASC. This discrepancy, regardless of differences between species, could arise from the cartilaginous articular environment, which may provide implanted cells with prochondrogenic stimuli, such as growth factors, low oxygen tension, and mechanical constraints [48]. These prochondrogenic stimuli are also known to favor MSC chondrogenesis and cartilage tissue maturation [49], [50]. In addition, the presence of progenitor cells in articular cartilage has been detected in the superficial zone of articular cartilage. Cell population CD105+/CD166+ exhibiting a high colony-forming efficiency, a chemotactic activity and limited multipotency has been described recently [51], [52], [53], [54]. These endogenous progenitors may influence the behavior of implanted cells and erase the differences observed after the preconditioning culture. However, the role and function of these endogenous progenitors have yet to be clearly deciphered, especially in the context of cartilage repair.

Altogether, the data obtained from the present rabbit and nude mice experiments demonstrate that although hypoxia strongly promotes the *in vitro* chondrogenic differentiation of ASC in a monolayer or entrapped within a Si-HPMC hydrogel, it fails to potentiate the formation of cartilaginous tissue *in vivo*. Viewing the discrepancy between the *in vitro* and *in vivo* data, it seems reasonable to speculate that cells implanted within Si-HPMC hydrogel experience some quite similar environmental factors, including low oxygen tension, that could greatly influence their ability to produce a cartilaginous tissue [30], [31]. The effects of this low oxygen tension are mainly to be mediated by the activation of the HIF transcription factor family [55]. As suggested by our *in vitro* data, such a low oxygen tension has indeed been reported to stimulate the chondrogenic differentiation through a specific stabilization of HIF1-alpha. It is well acknowledged that HIF-1 alpha/HIF-1 beta dimers upregulate the transcriptional activity of SOX9 promoter through binding on specific hypoxia-responsive element sequences [33], [56], which in turn increases the expression of type II collagen and aggrecan. In addition, it has been shown that low oxygen tension also contributes to the hydroxylation-mediated maturation of the collagen fibers through the increase in the expression of prolyl-4-hydroxylase [57], [58].

Regardless of the animal model used, one of the limitations in the present manuscript and in a large number of similar studies reported in the literature is the time point at which the formation of a repair tissue is investigated (18 weeks in rabbits and 5 weeks in nude mice). The maturation of the newly formed cartilage is indeed a complex, spatially- and temporally-regulated process that involved a large number of biological partners.

The Si-HPMC hydrogel that has long been considered a suitable vehicle for the delivery of cells in a cartilaginous defect via a minimally invasive surgical protocol should also be viewed as a permeable structure that allows cells to sense environmental prochondrogenic stimuli, such as growth factors, low oxygen tension and mechanical constraints.

Consequently, we hypothesized that the Si-HPMC hydrogel may provide a 3D scaffolding environment suitable for chondrogenesis. To address this issue, we cultured hASC in monolayers or within the Si-HPMC hydrogel under NCT, NCH and HCH conditions. Our results suggest that the *in vitro* 3D culture within the Si-HPMC hydrogel seems to enhance the prochondrogenic effects of the induction medium and hypoxia. Both these properties of our hydrogel are likely to make Si-HPMC a promising scaffolding biomaterial for MSC-based cartilage tissue engineering [10].

The successful regeneration of cartilage, however, requires that the cells be driven towards a stable articular phenotype, as opposed to a growth plate phenotype, which leads to hypertrophy and ultimately to cartilage calcification [59]. Five per cent oxygen has been shown to not only promote the chondrogenic differentiation of MSC, but also to prevent their hypertrophic differentiation [60], [61]. Thus, considering this effect of hypoxia on the terminal conversion of MSC towards a hypertrophic phenotype, testing whether hypoxic preconditioning of ASC could be used to prevent the formation of calcified tissue *in vivo* after long-term implantation remains of particular interest. This point will be addressed in future experiments.

## Conclusions

Our study shows that concomitant treatment with low oxygen tension and a chondrogenic medium promotes the *in vitro* chondrogenic differentiation of ASC of rabbit and human origin. In addition, our data indicate that the *in vitro* chondrogenic differentiation of ASC, regardless of oxygen preconditioning, is required for optimal cartilage regeneration *in vivo*. Although hypoxic preconditioning of ASC did not improve *in vivo* regeneration in our models, whether such preconditioning may help prevent the formation of calcified cartilage *in vivo* remains to be determined. These data together provide new insights into the biology of MSC and could help take advantage of their regenerative potential for the development of a clinically relevant cartilage tissue repair procedure.
